# The YTHDF1–TRAF6 pathway regulates the neuroinflammatory response and contributes to morphine tolerance and hyperalgesia in the periaqueductal gray

**DOI:** 10.1186/s12974-022-02672-y

**Published:** 2022-12-22

**Authors:** Handong Ouyang, Jianxing Zhang, Dongmei Chi, Kun Zhang, Yongtian Huang, Jingxiu Huang, Wan Huang, Xiaohui Bai

**Affiliations:** 1 Department of Anesthesiology, State Key Laboratory of Oncology in Southern China, Sun Yat-sen University Cancer Center, Collaborative Innovation Center for Cancer Medicine, 651 Dongfeng Rd East, Guangzhou, China; 2 Department of Anesthesiology, Guangdong Provincial Key Laboratory of Malignant Tumor Epigenetics and Gene Regulation, Sun Yat-sen Memorial Hospital, Sun Yat-sen University, 107 Yangjiang Road West, Guangzhou, China

**Keywords:** YTHDF1, TRAF6, Morphine tolerance, Morphine-induced hyperalgesia, Inflammatory factors

## Abstract

Long-term use of opioids such as morphine has negative side effects, such as morphine analgesic tolerance and morphine-induced hyperalgesia (MIH). These side effects limit the clinical use and analgesic efficacy of morphine. Elucidation of the mechanisms and identification of feasible and effective methods or treatment targets to solve this clinical phenomenon are important. Here, we discovered that YTHDF1 and TNF receptor-associated factor 6 (TRAF6) are crucial for morphine analgesic tolerance and MIH. The m6A reader YTHDF1 positively regulated the translation of TRAF6 mRNA, and chronic morphine treatments enhanced the m6A modification of TRAF6 mRNA. TRAF6 protein expression was drastically reduced by YTHDF1 knockdown, although TRAF6 mRNA levels were unaffected. By reducing inflammatory markers such as IL-1β, IL-6, TNF-α and NF-κB, targeted reduction of YTHDF1 or suppression of TRAF6 activity in ventrolateral periaqueductal gray (vlPAG) slows the development of morphine analgesic tolerance and MIH. Our findings provide new insights into the mechanism of morphine analgesic tolerance and MIH indicating that YTHDF1 regulates inflammatory factors such as IL-1β, IL-6, TNF-α and NF-κB by enhancing TRAF6 protein expression.

## Introduction

Chronic pain is a major public health issue that has a significant impact on people's quality of life [1]. For the management of refractory chronic pain, opioid analgesics such as morphine remain the gold standard. A widespread opioid crisis has resulted from the rise in the dosage used in opioid prescriptions over the last few decades [2, 3]. Chronic opioid usage can lead to analgesic tolerance, which is defined as a steady decline in analgesic effectiveness at fixed medication dosages, as well as paradoxical opioid-induced hyperalgesia (OIH) [4, 5]. The identification of various neural mechanisms implicated in opioid-induced tolerance and hyperalgesia has shown substantial progress in the last year [5]

Many studies have reported that periaqueductal gray (PAG) plays an important role in the effects of morphine, including morphine analgesia, morphine addiction and morphine withdrawal. Internally, the PAG is delineated by columnal-based boundaries: the dorsal PAG (dPAG), dorsolateral PAG (dlPAG), lateral PAG (lPAG) and ventrolateral PAG (vlPAG) [6, 7]. Different subregions of the PAG perform different functions, and the same type of cells in different PAG subregions perform different functions. Activating glutamatergic neurons in the vlPAG can induce freezing, but activating glutamatergic neurons in the dl/lPAG can inhibit freezing because glutamatergic neurons in the dl/lPAG can inhibit vlPAG glutamatergic neuron excitation by directly activating vlPAG GABAergic neurons [8]. We chose the vlPAG because it is an important brain region for analgesia tolerance. In vitro and in vivo studies have shown that the vlPAG is involved in regulating the development of opioid analgesic tolerance [9–11]. Tolerance rapidly develops following repeated administration of morphine into the vlPAG [12]. Blocking opioid binding in the vlPAG with the antagonist naltrexone significantly attenuates the development of tolerance to systemically administered morphine, indicating that key mechanisms underlying morphine tolerance (MT) are localized in the vlPAG [13]. Inhibition of G protein-activating enzymes in the vlPAG, such as adenylate cyclase (AC), c-Jun N-terminal kinase and protein kinase-c (PKC), can reduce the development of morphine tolerance [9, 14, 15]. Neuroinflammation in the vlPAG is involved in the development of morphine tolerance [16, 17]. Proinflammatory cytokines, including interleukin-1β (IL-1β), interleukin-6 (IL-6), tumor necrosis factor-α (TNF-α), and nuclear factor-κB (NF-κB), contribute to the development of morphine tolerance [18–21]. Understanding the mechanisms of opioid-induced neuroinflammation is valuable to develop effective pain management strategies. One possible effective method to reduce or eliminate opioid-induced analgesic tolerance and hyperalgesia is to reduce neuroinflammation in the vlPAG.

N6-Methyladenosine (m6A) is one of the most prevalent internal alterations of eukaryotic mRNA and impacts practically every stage of RNA metabolism, including splicing, degradation, output, and translation [22–24]. YTHDF1, YTHDF2, and YTHDF3 are three major “reader” proteins that have been proven to identify m6A nucleotides via their YTH (YT521-B homology) domain [24–26]. YTHDF1 has been shown to improve m6A-associated mRNA translational efficiency [24], whereas YTHDF2 has been shown to mediate m6A-associated mRNA instability [23]. More interestingly, YTHDF3 has been found to have a dual role, combining the features of YTHDF1 and YTHDF2 and depending on the m6A mRNA target [27, 28]. Recently, some studies have shown that m6A modification plays an important role in the central nervous system [29, 30] and is involved in regulating the development of neurons, such as proliferation and differentiation [31–33]. Other studies have shown that m6A modification plays a key role in the inflammatory response [34, 35]. However, it is unclear whether YTHDF1 can regulate opioid-induced neuroinflammation and tolerance.

TNF receptor-associated factor 6 (TRAF6) is widely involved in the inflammatory response and immune response mainly through inflammatory and apoptotic signaling pathways [36]. YTHDF1 plays an important role in the immune system by “reading” the m6A nucleotides in TRAF6 mRNA transcripts and directing their translation [34]. Inhibition of TRAF6 can reduce proinflammatory cytokines, including IL-1β, IL-6, and TNF-α, to alleviate the neuropathic pain caused by spinal nerve ligation (SNL) [37]. CircNf1 in the spinal cord functions as a sponge for miR-330-3p and miR-665, induces the upregulation of CXCL12 and contributes to morphine analgesic tolerance [38]. In addition, IL-33 participates in morphine tolerance and OIH through the TRAF6-JNK pathway [39]. Nevertheless, whether TRAF6 mediated by YTHDF1 is also involved in the formation of morphine tolerance and MIH is unclear. The hypothesis tested in the present study is that YTHDF1 in the vlPAG participates in morphine analgesic tolerance and MIH by regulating inflammatory factors such as IL-1β, IL-6, TNF-α and NF-κB involved in TRAF6. We hope to elucidate the mechanism of morphine analgesic tolerance and MIH using a completely novel pathway and provide a possible new therapeutic treatment target for morphine analgesic tolerance and MIH.

## Materials and methods

### Animals and ethical statement

Male C57/BL6 mice (8–10 weeks) were purchased from the Institute of Experimental Animals of Guangdong Medicine Experimental Animal Center. YTHDF1^flox/flox^ (YTHDF1^fl/fl^) mice were obtained from the laboratory of Prof. Ruihua Xu and Prof. Huaiqiang Ju as a gift. The mice were constructed by GemPharmatech (Nanjing, China), and the genotype was verified in Xu and Ju’s research [40]. We also verified all the YTHDF1^fl/fl^ mouse genotypes we used. All mice were housed in groups of five mice per cage under a standard 12-h light/dark cycle (light from 7:00 a.m. to 7:00 p.m.) at constant room temperature (25 ± 1 °C) with food and water available ad libitum. All experimental procedures were approved by the Use Committee of Sun Yat-sen University and Animal Care Committee and were conducted in accordance with the guidelines of the National Institutes of Health (NIH). All efforts were made to minimize the number of animals used as well as their suffering. Researchers were blinded to the mouse genotypes and drug treatment during the experiments.

### Chronic morphine treatments and behavior tests

All mice were habituated to the testing environment for 3 days. C57BL6 wild-type (WT) mice or YTHDF1^fl/fl^ mice were injected subcutaneously (s.c.) with morphine (Qing-hai Pharmaceutical Factory, Xining, China) (10 mg/kg) or saline (vehicle) twice daily (8 am and 5 pm) for 7 consecutive days.

### Hot plate analgesia assay

Analgesia was measured using a 52 °C hot plate apparatus (UGO Basile) as previously described [41]. Animals were habituated in the room for 1 h. For the MIH study, the baseline latencies to jump or lick the hind paw were measured. For the tolerance study, 30 min after morphine s.c., mice were placed on the hot plate, and the latencies to jump or lick the hind paw were recorded. A cutoff time of 45 s was used to avoid tissue damage and inflammation. The percent of maximum possible effect (%MPE) for morphine was calculated by the following formula: 100% × [(drug response latency - basal response latency)/(cutoff time - basal response latency)] = %MPE.

### Tail immersion assay

The tail immersion was assessed before and 30 min after morphine treatment as described previously [42]. In brief, the temperature of the water bath was set at 48 °C. Each mouse was gently introduced into a restrainer, and the protruding 2/3 end of its tail was dipped into a hot water bath. A positive response manifested as a reflexive withdrawal of the tail from the hot water, and the latency to this response was recorded. For the MIH study, thermal sensitivity was assessed before morphine s.c. For the tolerance study, 30 min after morphine s.c., mice tail response in hot water was recorded. A cutoff time of 25 s was chosen to prevent tissue damage. The %MPE calculation method is the same as that mentioned above.

### Adeno-associated virus (AVV) microinjection

Conditional knockdown of YTHDF1 expression in the vlPAG was achieved by bilateral stereotaxic injections of 150 nl of AVV-CMV-Cre-EGFP into the vlPAG of YTHDF1^fl/fl^ mice or a 150 nl mixture of AVV-CMV-Cre + AAV-CMV-DIO-EGFP-YTHDF1-shRNA into the vlPAG of the WT mice. The control animals received an injection of 150 nl of AAV-CMV-EGFP into the YTHDF1^fl/fl^ mice or 150 nl mixture of AAV-CMV-Cre + AAV-CMV-DIO-EGFP-YTHDF1-shRNA-negative control (NC) into the WT mice. Stereotaxic coordinates for viral vector injection were anteroposterior (AP),  − 4.45 mm; anterolateral (AL), ± 0.55 mm; and dorsoventral (DV), − 2.70 mm. For the virus microinjection, mice were anesthetized using isoflurane inhalation under an isothermal heating pad. The injection speed was adjusted to 15 nl/min under the control of a microinfusion pump. The pipette was kept in place for an additional 10 min after injection. The mice were allowed to recover for 3 weeks to allow stable transgene expression. All recombinant AAV was purchased from BrainVTA Technology Corporation (Wuhan, China).

### Drug infusions

For microinjection studies, mice were anesthetized under isoflurane anesthesia on an isothermal heating pad and securely placed into a stereotaxic device with the bregma and lambda horizontally level. A 30-gauge stainless-steel cannula with a 33-gauge stainless-steel stylet plug (RWD Life Science Co., Ltd, Shenzhen, China) was bilaterally implanted 0.5 mm above the vlPAG injection site anteroposterior (AP), − 4.45 mm; anterolateral (AL), ± 0.55 mm; and dorsoventral (DV), − 2.7 mm. The animals were allowed to recover for one week before the next experimental procedure. At the end of the experiment, brains were sectioned for cresyl violet staining to verify cannula position and injection site.

Mice were briefly anesthetized with isoflurane, and microinjection was performed through a 33-gauge stainless-steel injection cannula that extended 0.5 mm beyond the tip of the guide cannula. The injection cannula was connected to a 1 μL Hamilton syringe under the control of a microinfusion pump. C25-140 (0.01 mg/kg, 150 nl) (MCE, HY-120934) or the same volume of DMSO (150 nl) (MCE, USA) was injected over a 10-min period. The injection cannula was left in place for an additional 10 min to minimize spread of the drug along the injection track.

### Immunofluorescence

Mice were anesthetized using a sodium pentobarbital 50 mg/kg dose (i.p.).and perfused intracardially with saline and 4% paraformaldehyde (PFA) in 0.1 M PB. The vlPAG tissues were removed and post-fixed in 4% PFA overnight at 4 °C and transferred to 30% sucrose in 0.1 M PB at 4 °C for subsequent use. Then, vlPAG sections (25 μm, free-floating) were prepared using a cryostat and blocked with 10% normal donkey serum in 0.01 M PBS containing 0.3% Triton X-100 for 1 h at room temperature (approximately 26 °C). The vlPAG sections were then incubated overnight at 4 °C with primary antibodies against YTHDF1 (Proteintech, 17479-1-AP, 1:200), TRAF6 (Affinity Biosciences, AF5376, 1:200), IL-1β (Santa Cruz, sc-12742, 1:200), IL-6 (Affinity Biosciences, DF6087, 1:200), TNF-α (Boster, BA0131, 1:200) and NF-κB (Affinity Biosciences, AF0874, 1:200). The sections were then incubated for 1 h at room temperature with Cy3-conjugated secondary antibodies. The stained sections were examined using a Nikon confocal microscope, and images were captured with a Nikon DS-Qi2 camera.

### Western blot

Mouse brains were immediately removed and sectioned in cold oxygenated artificial cerebrospinal fluid after application of sodium pentobarbital at a 50 mg/kg dose (i.p.). The vlPAG tissues were punched using a 15-gauge cannula and homogenized in Tris containing proteinase and phosphatase inhibitors on ice. Proteins were separated by gel electrophoresis SDS-PAGE and transferred to PVDF membranes, which were then incubated with primary antibodies against YTHDF1 (Proteintech, 17479-1-AP, 1:1000), TRAF6 (Affinity Biosciences, AF5376, 1:1000), IL-1β (Santa Cruz, sc-12742, 1:1000), IL-6 (Affinity Biosciences, DF6087, 1:1000), TNF-α (Boster, BA0131, 1:1000), NF-κB (Affinity Biosciences, China, 1:1000) and GAPDH (Abcam, ab181602, 1:5000) overnight at 4 °C. The blots were then incubated with secondary antibodies conjugated to horseradish peroxidase. The immunostained bands were acquired by a computer-assisted chemiluminescence imaging analysis system (Tanon 5200, China). The intensities of the blots were quantified by densitometry. The blot density of the control rats was set at 100%. The relative density values of the other groups were determined by dividing the values for these groups by that of the controls.

### RNA extraction and quantitative polymerase chain reaction

TRIzol was used to extract total DNA from the vlPAG, and reverse transcription was performed following the protocol of the polymerase chain reaction (PCR) production kit (Accurate Biology, AG 11706). The following primer pairs were used for qRT-PCR: TRAF6 forward: AGGAATCACTTGGCACGACACTTG, reverse: CAGGGTCCGAATGGTCCGTTTG; GAPDH forward: AGGTCGGTGTGAACGGATTTG, reverse: TGTAGACCATGTAGTTGAGGTCA. The reaction cycle conditions were as follows: an initial denaturation at 95 °C for 3 min, followed by 40 thermal cycles of 10 s at 95 °C, 20 s at 58 °C, and 10 s at 72 °C. The ratio of mRNA expression in the vlPAG tissues was analyzed by the 2^−ΔΔCT^ method.

### m^6^A dot blot assay

Total RNA isolated from the vlPAG by TRIzol was mixed in three times the volume of incubation buffer and denatured at 65 °C for 5 min. Samples (200 ng, 100 ng, 50 ng) dissolved in SSC buffer were deposited on an Amersham Hybond-N + membrane (GE Healthcare, USA), which was settled on a Bio-Dot Apparatus. Then, the membrane was crosslinked by UV light for 5 min, followed by staining with 0.02% methylene blue. Scanning of blue dots was performed to show the input RNA content. The membrane was incubated with m6A antibody (Abcam, ab284130) overnight at 4 °C. Dot blots were acquired by a computer-assisted chemiluminescence imaging analysis system (Tanon 5200) after incubation with secondary antibodies conjugated to horseradish peroxidase.

### MeRIP-qPCR

The MeRIP assay was performed with the Magna MeRIP™ m6A KIT (Merck Millipore, 17-701) to determine the m6A medication. In brief, total RNA was extracted from mouse vlPAG. RNA samples were then immunoprecipitated with magnetic beads precoated with 10 μg anti-m6A antibody (Abcam, ab208577) or anti-mouse IgG according to the standard protocol of the Magna methylated RNA immunoprecipitation m6A Kit. The relative enrichment of m6A was normalized to the input: %Input = 1/10 × 2^Ct[IP] – Ct[input]^.

### Statistical analyses

SPSS 25.0 was used to analyze the data; the results are shown as the mean ± standard error of the mean (SEM). The data were analyzed using the two independent samples *t* test or repeated measures two-way ANOVA + Bonferroni or Dunnett post hoc test, as indicated in the main text or figure captions, as appropriate. All experiments were randomized and performed by a blinded researcher. Researchers remained blinded throughout the histological, biochemical and behavioral assessments.

## Results

### Chronic morphine treatments induce an inflammatory response and increase m6A methylation in the vlPAG

**Fig. 1:**
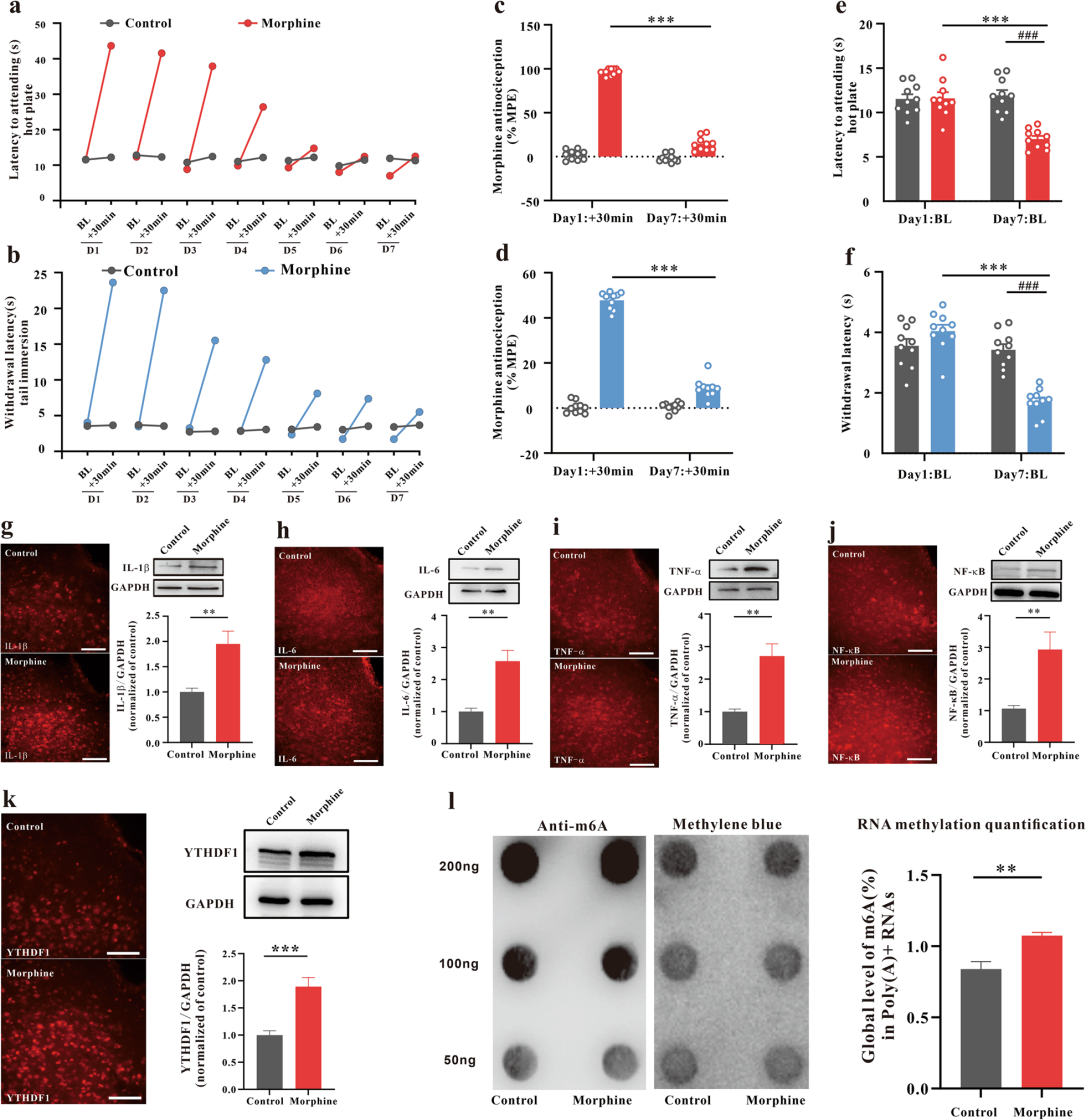
Chronic morphine treatments induce morphine analgesic tolerance and MIH, as well as an inflammatory response, m6A methylation and YTHDF1 in the vlPAG. **a**, **b** Time course of the daily hot-plate test and tail-immersion test before (baseline, BL) and after morphine treatment (+ 30 min) throughout a 7-d chronic morphine or saline exposure (10 mg/kg twice daily) in WT mice. Nociceptive behavior (pre-morphine BL time points only): hot plate, *F*_1,18_ = 18.094, *P* < 0.001; tail immersion, *F*_1,18_ = 7.472, *P* = 0.014. Antinociception (post-morphine + 30 min time points only): hot plate, *F*_1,18_ = 700.978, *P* = 0.001; tail immersion, *F*_1,18_ = 465.072, *P* < 0.001. **c**, **d** Antinociception tolerance. Percentage of the maximal possible effect (%MPE) for morphine antinociception from the first administration (Day 1: + 30 min) compared with the last administration (Day 7: + 30 min) (hot plate, *F*_1,18_ = 809.972, *P* < 0.001. ***P < 0.001 vs. Day 1 + 30 min; tail immersion, *F*_1,18_ = 446.943*, P* < 0.001. ***P < 0.001 vs. Day 1 + 30 min). **e**, **f** MIH. Withdrawal latency change before and after morphine administration (hot plate: *F*_1,18_ = 14.677, *P* = 0.001. ***P < 0.001 vs. Day 1 BL of the MT group, ^###^ vs. Day 7 BL of the control groups; tail immersion: *F*_1,18_ = 63.115, *P* < 0.001. ***P < 0.001 vs. Day 1 BL of the MT group, ^###^ vs. Day 7 BL of the control groups) (repeated measures two-way ANOVA, n = 10). **g–k** Immunofluorescence images and western blotting analysis of IL-1β (**g**, *P* = 0.00504), IL-6 (**h**, *P* = 0.00117), TNF-α (**i**, *P* = 0.00132), NF-κB (**j**, *P* = 0.0076), and YTHDF1 (**k**, *P* = 0.00073) in the vlPAG of the mice treated with repetitive saline or morphine administration (n = 6), Student’s *t* test, two-tailed. **l** The m6A of poly (A) + isolated from total RNA of the vlPAG after a 7-d morphine or saline treatments was indicated by m6A dot blot. Corresponding RNAs were loaded equally by twofold serial dilution with 200 ng, 100 ng, and 50 ng. Methylene blue staining served as a loading control (*P* = 0.0065, n = 4, Student’s *t* test, two-tailed). Tissues were collected on Day 7 after administration. Scale bars, 50 μm. Data are shown as the mean ± SEM

To explore whether m6A methylation affects the inflammatory response of the vlPAG in chronic morphine treatments, we first investigated m6A levels in a model of morphine analgesic tolerance and OIH. After chronic morphine exposure in mice (injected subcutaneously with 10 mg/kg, twice daily for 7 days), antinociceptive tolerance and thermal hyperalgesia were induced. As shown in Fig. 1a and b, repeated morphine treatment produced a progressive and striking decline in morphine analgesic efficacy over a 7-day test period in the hot plate and tail immersion tests in WT mice. We compared the percent of maximum possible effect (%MPE) between the morphine group and the saline group (Day 7 vs. Day 1) to evaluate analgesic tolerance (Fig. 1c, d). In addition, we compared the withdrawal latency (Day 7 vs. Day 1) at baseline between the morphine group and saline group to evaluate the hyperalgesia induced by continuous use of morphine (Fig. 1e, f). Chronic morphine treatments increased the protein expression of IL-1β, IL-6, TNF-α and NF-κB measured by western blots and subjective immunofluorescence intensity in the vlPAG (Fig. 1g-h). We also investigated the expression patterns of m6A readers (YTHDF1) in the model of morphine analgesic tolerance and thermal hyperalgesia (Fig. 1k). Compared to the control, chronic morphine treatments increased the m6A levels (Fig. 1l). These data suggested that inflammation, m6A methylation and the m6A reader protein YTHDF1 may participate in the pathological process of morphine analgesic tolerance and thermal hyperalgesia.

### vlPAG YTHDF1 expression mediates analgesic tolerance and MIH

**Fig. 2:**
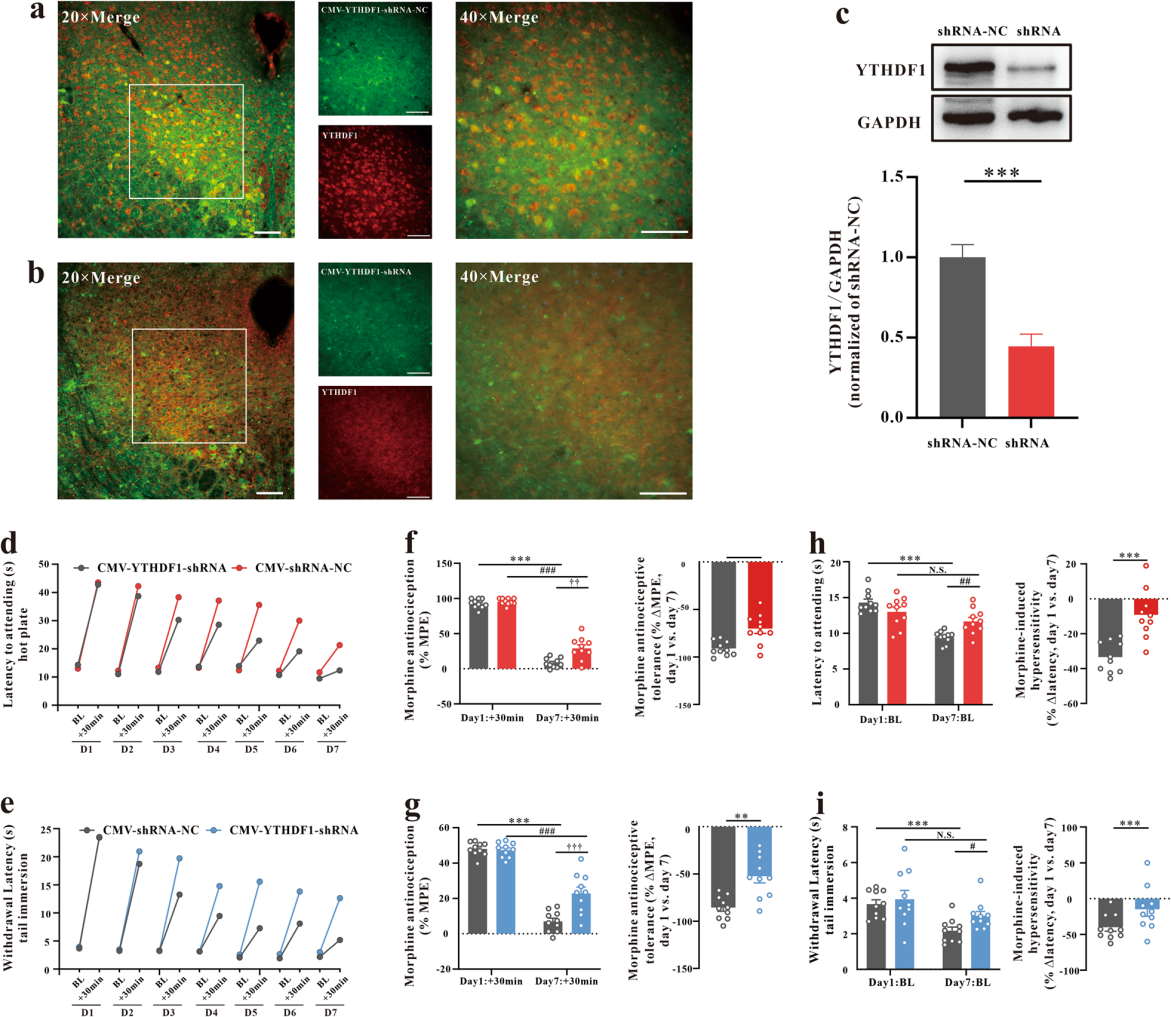
Knocking down YTHDF1 expression in vlPAG by YTHDF1-shRNA decreased analgesic tolerance and MIH. **a**, **b** Immunostaining verifying YTHDF1 downregulation in the vlPAG of CMV-YTHDF1-shRNA-NC or CMV-YTHDF1-shRNA in WT mice. Scale bars, 50 μm. **c** Western blotting analysis verifying YTHDF1 downregulation in the vlPAG of CMV-YTHDF1-shRNA in WT mice (*P* = 0.0005, n = 6, Student’s t test, two-tailed). **d**, **e** Nociceptive behavior (BL time): hot plate, *F*_1,18_ = 0.553, *P* = 0.467; tail immersion, *F*_1,18_ = 2.158, *P* = 0.159. Antinociception: hot plate, *F*_1,18_ = 17,546, *P* = 0.001; tail immersion, *F*_1,18_ = 14.946, *P* = 0.001. **f**, **g** Morphine antinociception (%MPE). **f** Hot plate, left, *F*_1,18_ = 11.74, *P* = 0.003. *** *P* < 0.001 vs. Day 1 + 30 min of the shRNA-NC group; ^###^ *P* < 0.001, vs. Day 1 + 30 min of the YTHDF1-shRNA group; †† *P* = 0.00126, vs. Day 7 + 30 min of the shRNA-NC group; right, the percent change for each subject, ** *P* = 0.00151 vs. the shRNA-NC group; **g** Tail immersion, left, *F*_1,18_ = 18.776, *P* < 0.0001. *** *P* < 0.001 vs. Day 1 + 30 min of the shRNA-NC group; ^###^ *P* < 0.001, vs. Day 1 + 30 min of the YTHDF1-shRNA group; †† *P* = 0.001, vs. Day 7 + 30 min of the shRNA-NC group; right, the percent change for each subject, *** *P* < 0.001 vs. the shRNA-NC group; **h**, **i** Changes in withdrawal latency before and after morphine administration. **h** Hot plate: left, *F*_1,18_ = 20.108, ***P < 0.001 vs. Day 1 BL of the shRNA-NC group; N.S.* P* = 0.1422 vs. Day 1 BL of the YTHDF1-shRNA group; ^##^*P* = 0.00246, vs. Day 7 BL of the shRNA-NC group; right, the percent change for each subject, ***P < 0.001 vs. the shRNA-NC group; **i** Tail immersion: left, *F*_1,18_ = 24.516,* P* < 0.001. ***P < 0.001 vs. Day 1 BL of the shRNA-NC group; N.S.* P* = 0.1095 vs. Day 1 BL of the YTHDF1-shRNA group; ^#^*P* = 0.0197, vs. Day 7 BL of shRNA-NC group; right, the percent change for each subject, *** *P* < 0.001 vs. shRNA-NC group; (repeated measures two-way ANOVA, n = 10) (Data are shown as the mean ± SEM. %MPE = percent of maximal possible effect; N.S. = No significance; CMV-YTHDF1-shRNA-NC = AAV-CMV-Cre + AAV-CMV-DIO-EGFP-YTHDF1-shRNA-NC; CMV-YTHDF1-shRNA = AAV-CMV-Cre + AAV-CMV-DIO-EGFP-YTHDF1-shRNA)

**Fig. 3:**
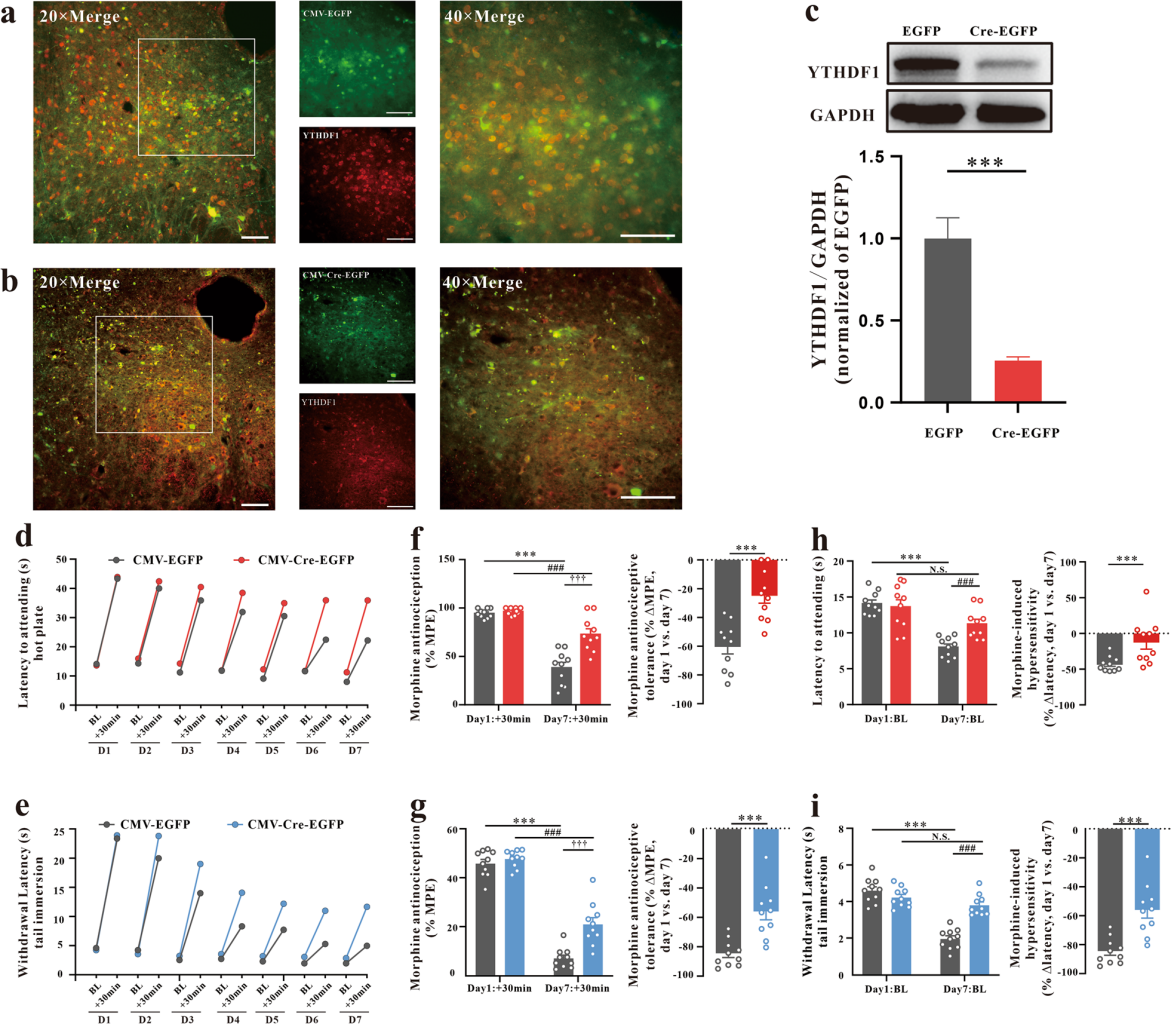
Knockdown of YTHDF1 in the vlPAG by CMV-Cre-EGFP in YTHDF1^fl/fl^ mice decreased morphine analgesic tolerance and MIH. **a**, **b** Immunostaining verifying YTHDF1 downregulation in the vlPAG of CMV-Cre-EGFP in YTHDF1^fl/fl^ mice. Scale bars, 50 μm. **c** Western blotting analysis verifying YTHDF1 downregulation in the vlPAG of CMV-Cre-EGFP in YTHDF1^fl/fl^ mice (***P < 0.001, n = 6, Student’s t test, two-tailed). **d**, **e** Nociceptive behavior (BL time): hot plate, *F*_1,18_ = 4.87, *P* = 0.041; tail immersion, *F*_1,18_ = 20.69, *P* < 0.001. Antinociception: hot plate, *F*_1,18_ = 15.576, *P* = 0.001; tail immersion, *F*_1,18_ = 22.084, *P* < 0.001. **f–g** Morphine antinociception (%MPE). **f** Hot plate, left, *F*_1,18_ = 21.037, *P* < 0.0001; ***P < 0.001 vs. Day 1 + 30 min of the CMV-EGFP group; ^###^*P* < 0.001, vs. Day 1 + 30 min of the CMV-Cre-EGFP group; †††*P* < 0.001, vs. Day 7 + 30 min of the CMV-EGFP group; right, the percent change for each subject, ***P < 0.001 vs. the CMV-EGFP group; **g** Tail immersion, left, *F*_1,18_ = 11.197, *P* = 0.004; ***P < 0.001 vs. Day 1 + 30 min of the CMV-EGFP group; ^###^*P* < 0.001, vs. Day 1 + 30 min of CMV-Cre-EGFP group; †††*P* < 0.001, vs. Day 7 + 30 min of CMV-EGFP group; right, the percent change for each subject, ***P < 0.001 vs. the CMV-EGFP group; **h**, **i** changes in withdrawal latency before and after morphine administration. **h** Hot plate: left, *F*_1,18_ = 33.564,* P* < 0.001; ***P < 0.001 vs. Day 1 BL of the CMV-EGFP group; N.S.* P* = 0.0599 vs. Day 1 BL of the CMV-Cre-EGFP group; ^###^*P* < 0.001, vs. Day 7 BL of the CMV-EGFP group; right, the percent change for each subject, **P = 0.00962 vs. the CMV-EGFP group; **i** tail immersion: left, *F*_1,18_ = 67.778,* P* < 0.001; ***P < 0.001 vs. Day 1 BL of the CMV-EGFP group; N.S.* P* = 0.1045 vs. Day 1 BL of the CMV-Cre-EGFP group; ^###^*P* < 0.001, vs. Day 7 BL of the CMV-EGFP group; right, the percent change for each subject, ***P < 0.001 vs. the CMV-EGFP group; (repeated measures two-way ANOVA, n = 10) (Data are shown as the mean ± SEM. %MPE = percent of maximal possible effect; N.S. = No significance)

Then, we explored the action of YTHDF1 under morphine-induced analgesic tolerance and MIH. AAV-CMV-Cre-EGFP with AAV-CMV-DIO-YTHDF1-shRNA or AAV-CMV-DIO-YTHDF1-shRNA-NC virus was injected into the vlPAG of WT mice (Fig. 2a–c). Knockdown of YTHDF1 in the vlPAG attenuated chronic morphine-induced analgesic tolerance and thermal hyperalgesia (Fig. 2d–i). To further verify the role of YTHDF1, AAV-CMV-Cre-EGFP or an AAV-CMV-EGFP control virus was injected into the vlPAG of YTHDF1^fl/fl^ mice. Three weeks later, when maximal viral expression was achieved (Fig. 3a–c), we measured latency to attending the hot plate and withdrawal latency of tail immersion before and 30 min after morphine injection each morning to evaluate analgesic tolerance and MIH, respectively (Fig. 3d–i). Notably, the knockdown of YTHDF1 in the vlPAG alleviated morphine analgesic tolerance and MIH.

### YTHDF1 regulates morphine-induced inflammation in the vlPAG

**Fig. 4:**
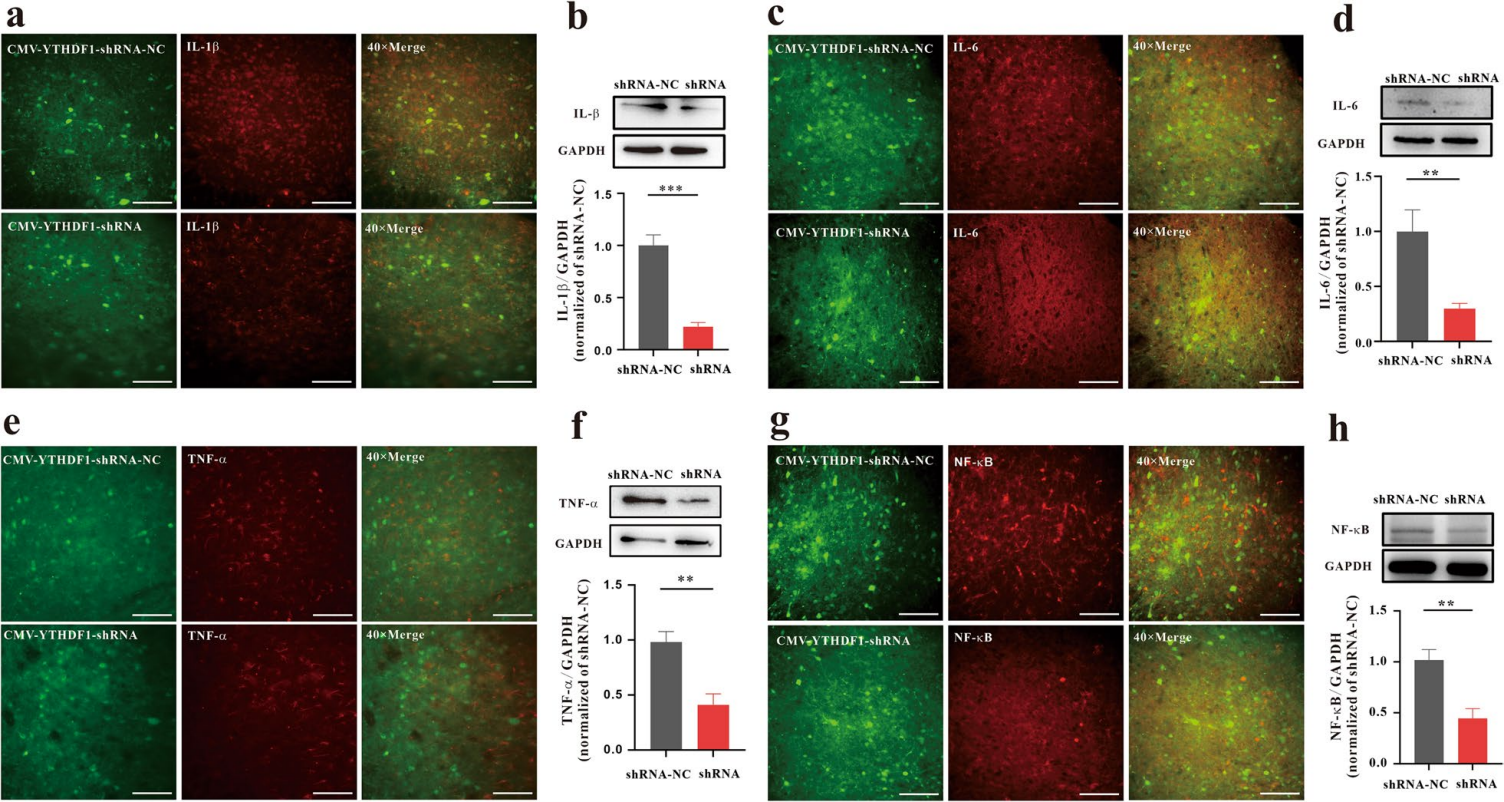
YTHDF1 regulates morphine-induced inflammation in the vlPAG. Knockdown of YTHDF1 in the vlPAG by CMV-YTHDF1-shRNA in WT mice inhibited the expression (IHC) of inflammatory factors such as IL-1β (**a**), IL-6 (**c**) TNF-α (**e**), and NF-κB (**g**). Scale bars, 50 μm. **b**, **d**, **f**, **h** Knockdown of YTHDF1 in the vlPAG by shRNA in virus-infected WT mice inhibited the expression (western blotting) of inflammatory factors such as IL-1β (**b**, ***P < 0.001), IL-6 (**d**, **P = 0.00612), TNF-α (**f**, **P = 0.00209) and NF-κB (**h**, **P = 0.0023) (n = 6, Student’s t test, two-tailed. Data are shown as the mean ± SEM; CMV-YTHDF1-shRNA-NC = AAV-CMV-Cre + AAV-CMV-DIO-EGFP-YTHDF1-shRNA-NC; CMV-YTHDF1-shRNA = AAV-CMV-Cre + AAV-CMV-DIO-EGFP-YTHDF1-shRNA)

**Fig. 5:**
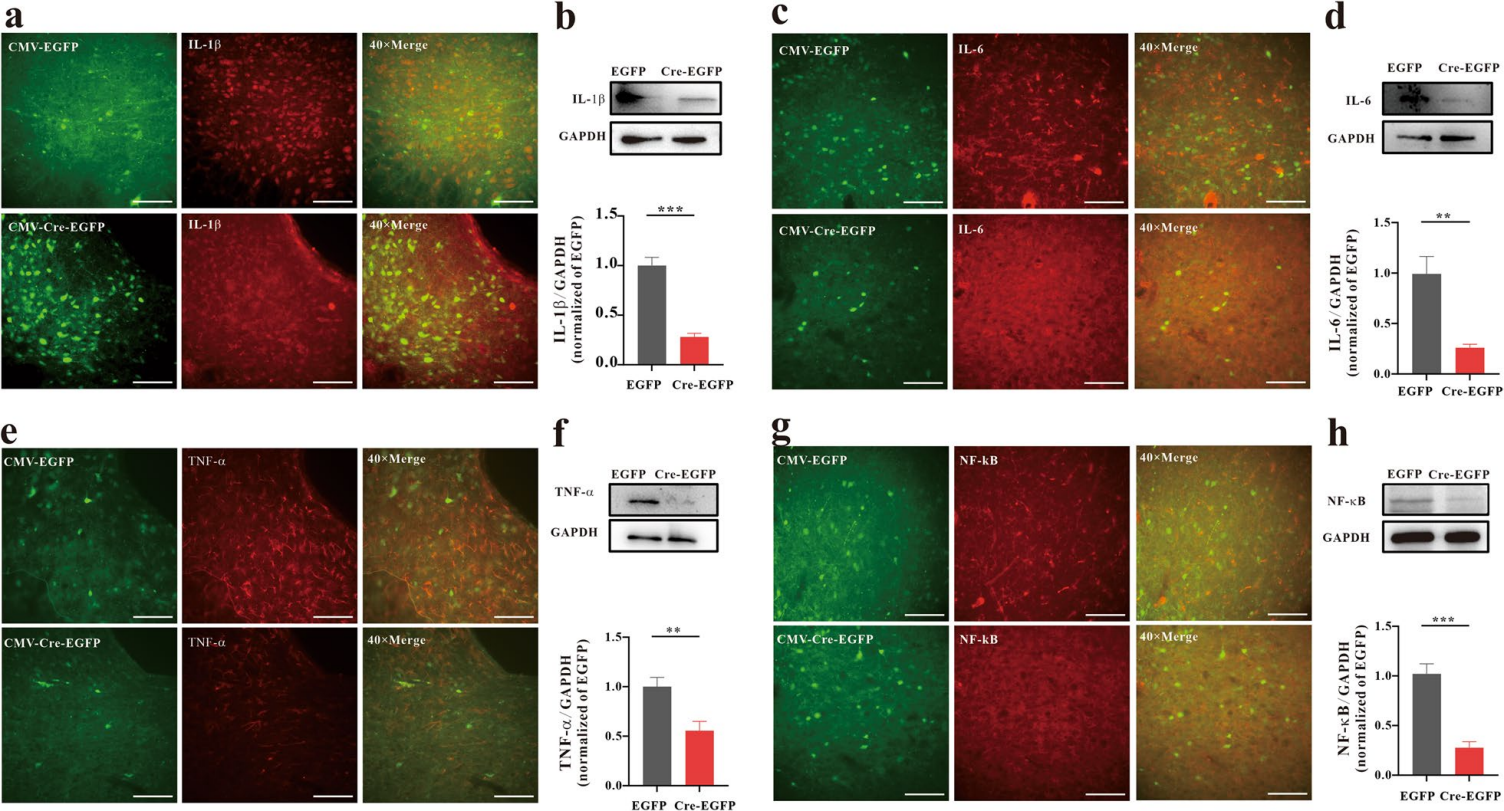
YTHDF1 regulates morphine-induced inflammation in the vlPAG. Knockdown of YTHDF1 in the vlPAG by CMV-Cre-EGFP in YTHDF1^fl/fl^ mice inhibited the expression (IHC) of inflammatory factors such as IL-1β (**a**), IL-6 (**c**) TNF-α (**e**), and NF-κB (**g**). Scale bars, 50 μm. **b**, **d**, **f**, **h** Knockdown of YTHDF1 in the vlPAG by CMV-Cre-EGFP in YTHDF1^fl/fl^ mice inhibited the expression (western blotting) of inflammatory factors such as IL-1β (**b**, ***P < 0.001), IL-6 (**d**, **P = 0.00202), TNF-α (**f**, **P = 0.00749) and NF-κB (**h**, ***P < 0.001) (n = 6), Student’s t test, two-tailed. Data are shown as the mean ± SEM

Given the upregulation of YTHDF1 protein expression in morphine analgesic tolerance and MIH, we next verified whether YTHDF1 reduces the inflammatory response in morphine analgesic tolerance and MIH. As above, we knocked down YTHDF1 in the vlPAG in WT mice or YTHDF1^fl/fl^ mice. IHC and western blotting results showed that the expression of inflammation-related factors, such as IL-1β, IL-6, TNF-α and NF-κB, decreased significantly after YTHDF1 knockdown (Figs. 4, 5). Therefore, we speculate that YTHDF1 may contribute to the mechanism of morphine-induced tolerance and MIH by regulating inflammatory-related factor expression.

### TRAF6 in the vlPAG is involved in morphine analgesic tolerance and MIH

**Fig. 6:**
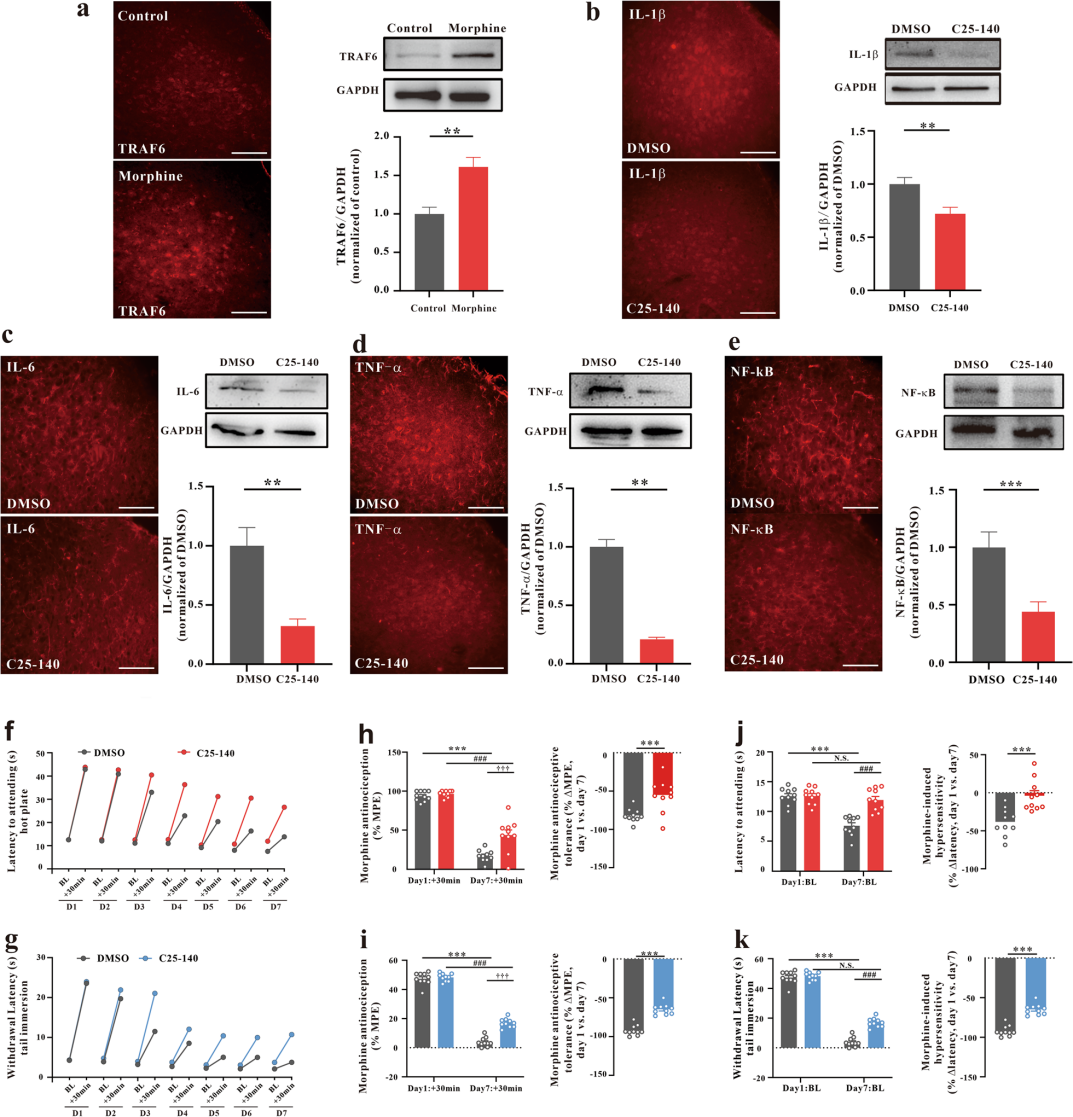
Inhibition of TRAF6 attenuated the chronic morphine treatment-mediated inflammatory response and reduced morphine tolerance and MIH. **a** Immunostaining and western blotting verified that chronic morphine treatments increased TRAF6 expression in the vlPAG (**P = 0.0025) (n = 6, Student’s *t* test, two-tailed). **b–e** IHC and western blotting assays showed that inhibition of TRAF6 in the vlPAG by C25-140 in WT mice decreased the expression of inflammatory factors, such as IL-1β (**b**, ** *P* = 0.00976), IL-6 (**c**, **P = 0.00214), TNF-α (**d**, ***P < 0.001) and NF-κB (**e**, **P = 0.00586), (n = 6, Student’s t test, two-tailed). **f**, **g** Nociceptive behavior (BL time): hot plate, *F*_1,18_ = 28.552, *P* < 0.001; tail immersion, *F*_1,18_ = 10.162, *P* = 0.005. Antinociception: hot plate, *F*_1,18_ = 35.82, *P* < 0.001; tail immersion, *F*_1,18_ = 77.046, *P* < 0.001. **h**, **i** Morphine antinociception (%MPE). **h** Hot plate, left, *F*_1,18_ = 10.447, *P* = 0.005. ***P < 0.001 vs. Day 1 + 30 min of the DMSO group; ^###^*P* < 0.001, vs. Day 1 + 30 min of the C25-140 group; †††*P* < 0.001, vs. Day 7 + 30 min of the DMSO group; right, the percent change for each subject, **P = 0.00201 vs. the DMSO group; **i** tail immersion, left, *F*_1,18_ = 35.75, *P* < 0.0001; *** *P* < 0.001 vs. Day 1 + 30 min of the DMSO group; ^###^ *P* < 0.001, vs. Day 1 + 30 min of the C25-140 group; †††*P* < 0.001, vs. Day 7 + 30 min of the DMSO group; right, the percent change for each subject, ***P < 0.001 vs. the DMSO group; **j**, **k** changes in withdrawal latency before and after morphine administration. **j** Hot plate: left, *F*_1,18_ = 24.497,* P* < 0.001. ***P < 0.001 vs. Day 1 BL of the DMSO group; N.S.* P* = 0.3805 vs. Day 1 BL of the C25-140 group; ^###^*P* < 0.001, vs. Day 7 BL of the DMSO group; right, the percent change for each subject, ***P < 0.001 vs. the DMSO group; **k** tail immersion: left, *F*_1,18_ = 56.382,* P* < 0.001; ***P < 0.001 vs. Day 1 BL of the DMSO group; N.S.* P* = 0.0715 vs. Day 1 BL of the C25-140 group; ^###^*P* < 0.001, vs. Day 7 BL of the DMSO group; right, the percent change for each subject, ***P < 0.001 vs. the DMSO group; (repeated measures two-way ANOVA, n = 10). (Data are shown as the mean ± SEM. %MPE = percent of maximal possible effect; N.S. = no significance.)

TRAF6 is a critical signaling transducer in morphine-induced tolerance and hyperalgesia in the spinal cord [39]. C25-140, a first-in-class and fairly selective TRAF6-Ubc13 inhibitor, directly binds to TRAF6 and blocks the interaction of TRAF6 with Ubc13. C25-140 decreases TRAF6 activity, reduces NF-κB activation, and combats autoimmunity [43, 44]. This molecule can inhibit the release of inflammatory factors such as IL-1β, IL-6, TNF-α and NF-κB by binding TRAF6 [44, 45]. Thus, we continued to verify whether TRAF6 in the vlPAG was involved in morphine analgesic tolerance and MIH. First, we analyzed changes in the protein expression of TRAF6 during chronic morphine treatments (Fig. 6a). The TRAF6 inhibitor C25-140 was injected into the vlPAG during chronic morphine treatments. WB and IHC assays were performed to detect the inflammatory response. We found that C25-140 inhibited IL-1β, IL-6, TNF-α and NF-κB expression in the morphine analgesic tolerance model (Fig. 6b–e). Furthermore, C25-140 also alleviated morphine analgesic tolerance and MIH (Fig. 6f–k). The aforementioned results suggest that inhibition of TRAF6 attenuates morphine analgesic tolerance and MIH by attenuating the expression of IL-1β, IL-6, TNF-α and NF-κB.

### YTHDF1 regulates the expression of TRAF6 in the vlPAG during morphine analgesic tolerance and MIH

**Fig. 7:**
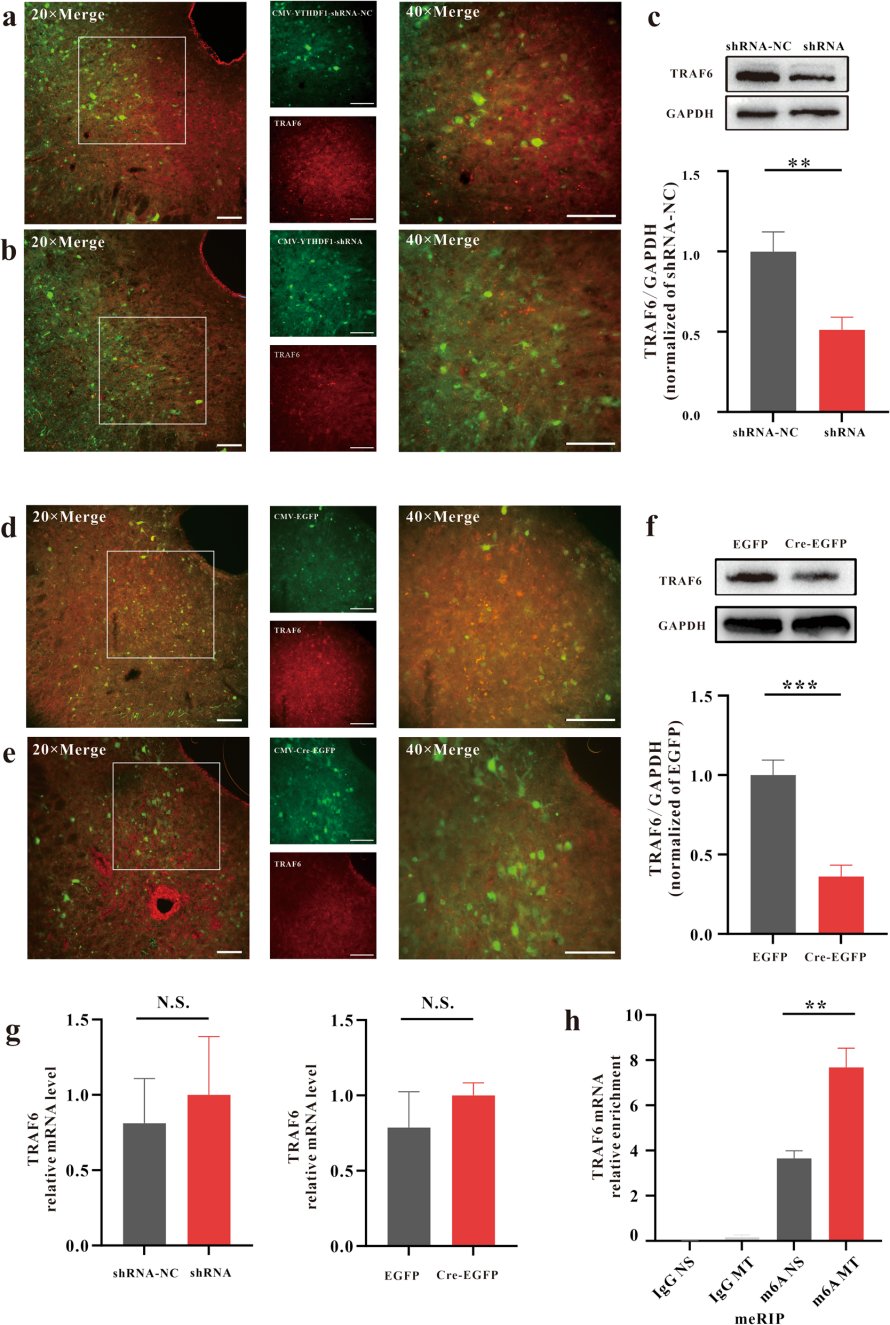
YTHDF1 regulates the expression of TRAF6 in the vlPAG during morphine analgesic tolerance and MIH. Immunostaining verifying that knockdown of YTHDF1 in the vlPAG by CMV-YTHDF1-shRNA in WT mice (**a–b**) or CMV-Cre-EGFP in YTHDF1^fl/fl^ mice (**d–e**) can inhibit the expression of TRAF6. Scale bars, 50 μm. **c**, **f** Western blotting analysis verifying that knockdown of YTHDF1 in the vlPAG by CMV-YTHDF1-shRNA in WT mice (**c**, **P = 0.00738, n = 6)) or CMV-Cre-EGFP in YTHDF1^fl/fl^ mice (**f**, ***P < 0.0001, n = 6) inhibited the expression of TRAF6. **g** Summary of *Traf6* mRNA measured in the vlPAG by chronic morphine treatments expressing CMV-YTHDF1-shRNA-NC or CMV-YTHDF1-shRNA, CMV-EGFP or CMV-Cre-EGFP relative to GAPDH mRNA (left, N.S. *P* = 0.430, n = 4; right, N.S. *P* = 0.7133, n = 4). **h** MeRIP-qPCR analysis of the m6A levels of TRAF6 mRNA in vlPAG with chronic saline or morphine treatments. (**P = 0.00133 vs. the control group) (n = 6, Student’s *t* test, two-tailed. Data are shown as the mean ± SEM, N.S. = no significance; CMV-YTHDF1-shRNA-NC = AAV-CMV-Cre + AAV-CMV-DIO-EGFP-YTHDF1-shRNA-NC; CMV-YTHDF1-shRNA = AAV-CMV-Cre + AAV-CMV-DIO-EGFP-YTHDF1-shRNA)

YTHDF1 was shown to modulate the translation of TRAF6 to mediate the intestinal immune response by m6A methylation [34]. We wanted to determine whether the YTHDF1/TRAF6 pathway plays an important role in the morphine analgesic tolerance model. As above, we knocked down YTHDF1 expression in the vlPAG in WT or YTHDF1^fl/fl^ mice. We found that the expression of TRAF6 also decreased significantly after YTHDF1 knockdown (Fig. 7a–f). However, the mRNA level of TRAF6 did not significantly change (Fig. 7g). Moreover, we performed m6A-IP qPCR and found that the m6A levels of TRAF6 mRNA were significantly increased in the morphine-induced analgesic tolerance model (Fig. 7h). Collectively, these results indicate that YTHDF1 regulates TRAF6 expression involved in morphine analgesic tolerance and MIH. The specific regulatory mechanism may be that THDHF1 recognizes the m6A modification site of TRAF6 mRNA and promotes downstream target gene translation.

## Discussion

The study provided new insight into the pathogenesis of morphine analgesic tolerance and MIH. The prolonged use of morphine can decease analgesic efficacy and lead to rapid morphine analgesic tolerance and cause a paradoxical hypersensitivity named morphine-induced hyperalgesia, which drives dose escalation and limits its clinical usage. Among the many epigenetic modification studies, the m6A modification in RNA has been shown to play a pivotal role in the central nervous system [29, 30]. However, the specific functional role of the m6A “reader” protein YTHDF1 in morphine analgesic tolerance and the MIH process remains unclear. Our study shows that YTHDF1 affects inflammatory-related factors, such as IL-1β, IL-6, TNF-α and NF-κB, by regulating the expression of TRAF6 and then participates in the regulation of morphine analgesic tolerance and MIH.

The cellular and molecular mechanisms of morphine analgesic tolerance and MIH have been comprehensively reviewed [46–48]. The mechanism leading to MIH may involve the NMDA glutamate system and transient receptor potential channels V1 and M8 (TRPV1 and TRPM8) and may be affected by a variety of factors, including genetic background and sex differences in experimental animals [49]. In addition, some reviews indicate that morphine analgesic tolerance and MIH may have a common pathway, such as opioid μ receptor signaling, pronociceptive ion channels, microglia, transcriptional mechanisms and inflammatory cytokines [5, 50]. Intrathecal injection of an IL-1 receptor antagonist in the spinal cord can attenuate both morphine analgesic tolerance and MIH in rats [51]. Inhibition of HMGB1 attenuates both morphine analgesia tolerance and MIH [52]. The release of inflammatory-related factors such as IL-1β, IL-6, TNF-α and NF-κB caused by chronic morphine treatments may be one of the pathogenic mechanisms of morphine analgesic tolerance and OIH [16, 52, 53]. Inhibition of TNF-α in animal experiments can alleviate the development of morphine analgesic tolerance [54]. The development of morphine analgesic tolerance can be significantly reduced by inhibiting IL-1β signaling [55, 56]. Inhibition of the NF-κB signaling pathway can significantly alleviate morphine analgesic tolerance and MIH [21, 52]. Regarding IL-6, some studies have shown that inhibition of IL-6 can alleviate morphine analgesic tolerance [19]. In addition, IL-6 knockout mice can significantly alleviate the adverse reactions caused by long-term morphine treatments [57]. Understanding how the body controls the intensity of the secretion of inflammatory factors is a key focus to understand the mechanism of morphine analgesic tolerance and OIH [46, 58]. This study also confirmed that chronic morphine treatments upregulate proinflammatory factors such as IL-1β, IL-6, TNF-α and NF-κB in the vlPAG by western blotting and IHC. Therefore, proinflammatory factors in the vlPAG may be involved in the formation of morphine analgesic tolerance and MIH.

We further investigated the regulatory mechanisms of YTHDF1 on morphine analgesic tolerance and MIH and confirmed that YTHDF1 was capable of regulating the inflammatory response in vlPAG-induced chronic morphine treatments. At the biological level, m6A-containing nucleotides are functionally medicated by the coordinated activity of methyltransferases, demethylases, and reader proteins [59]. The m6A reader protein YTHDF1 has been proven to be involved in the inflammatory response. This protein can not only promote the inflammatory response by regulating the translation of P65 or NLRP3 [60, 61], but also inhibit the inflammatory response through SOCS3 [62]. Inhibiting YTHDF1 expression in the infralimbic cortex attenuated remote fear extinction retention by decreasing NR2B and GluR1 expression and modulating dendritic spine remodeling [63]. YTHDF1 regulates axonal guidance by controlling the expression of Robo3.1 in the spinal cord through translation [64]. We delivered AAV-CMV-Cre-EGFP and AAV-CMV-Cre + AAV-CMV-DIO-EGFP-YTHDF1-shRNA to knock down YTHDF1 expression in YTHDF1^fl/fl^ mice and WT mice, respectively. Our results showed that these two knockdown methods effectively inhibited the YTHDF1 upregulation, TRAF6 expression and the downstream inflammatory-related factors caused by chronic morphine treatment. Different kinds of cells in the nervous system exert different effects on the inflammatory response. In addition to neurons, microglia and astrocytes are involved in many inflammatory reactions and the release of inflammatory factors [65–67]. Inflammation caused by microglia and astrocytes also contributed to the mechanism of morphine-induced tolerance and MIH. Chronic morphine exposure results in a strong upregulation of the microglial markers CD1 1b and Iba1, as well as the ATP receptors P2X4 and P2X7 in spinal microglia, and inhibition of p38 activation in spinal microglia prevents the development of morphine tolerance [68–70]. Chronic morphine treatment upregulated the expression of the cytokine interleukin-33 (IL-33) primarily in oligodendrocytes and astrocytes and that of its receptor ST2 mainly in astrocytes [39]. Inhibition of IL-33 or ST2 attenuates morphine tolerance by reducing morphine-enhanced astrocyte activation, excitatory synaptic transmission and CXCL12 expression [39]. Recently, YTHDF1 and m6A modification were reported to be involved in the inflammatory response of microglia [60]. These results indicate that YTHDF1 may also play a role in regulating the function of microglia. In our research, to obtain the maximum extent of YTHDF1 knockdown effectiveness, we used the strong nonselective cell promoter cytomegalovirus (CMV) to globally knock down YTHDFF1. We cannot rule out the distinct role of YTHDF1 in different types of cells in morphine analgesic tolerance and MIH, which requires further research. However, our research results still show that YTHDF1 silencing could inhibit the secretion of inflammatory factors, including IL-1β, IL-6, TNF-α and NF-κB, in the vlPAG, suggesting that YTHDF1 may be one of the key factors regulating the inflammatory response to chronic morphine treatments. Knocking down YTHDF1 delayed the development of morphine analgesic tolerance and MIH. To date, no evidence has shown that morphine can directly activate and upregulate YTHDF1. Only a few studies have explored the regulation of YTHDF1 expression. Hypoxia [71], c-Myc [72], the Wnt/β-catenin signaling pathway [73], the m6A “eraser” ALKBH5 [74], and miR-3436 [75] were reported to regulate YTHDF1 expression in cancer research. Our recent research shows that activation of the WNT3a pathway can promote the expression of YTHDF1 in pathological pain caused by oxaliplatin, and inhibition of the WNT3a pathway can downregulate the expression of YTHDF1 [76]. Morphine exposure can cause the upregulation of WNT3a [77, 78]. We speculate that morphine may cause the upregulation of YTHDF1 through the WNT3a signaling pathway. There may be another pathway by which morphine regulates YTHDF1 expression, and this requires further research.

Previous studies have shown that injection of TRAF6 siRNA can reduce neuropathic pain and inflammatory pain behavior by reducing the inflammatory response caused by spinal nerve ligation or neonatal colitis [37, 79–81]. TRAF6 is involved in the expression and secretion of the inflammatory cytokines IL-1β, IL-6, TNF-α and NF-κB [34, 37, 39]. The increase in p-JNK expression induced by chronic morphine treatments can be significantly inhibited by knocking down TRAF6 in the spinal cord and delaying morphine analgesic tolerance and hyperalgesia [39]. Our results found that TRAF6 increased in the vlPAG in morphine analgesic tolerance and MIH. Similarly, we found that inhibition of TRAF6 activity by an inhibitor of C25-140 could alleviate morphine analgesic tolerance and MIH by inhibiting the release of inflammatory factors such as IL-1β, IL-6, TNF-α and NF-κB in the vlPAG. In the intestinal immune response to bacterial infection, YTHDF1 can facilitate the immune inflammatory response by regulating the transcription of TRAF6 [34]. Our results showed that chronic morphine treatments increased the m6A levels in the vlPAG (Fig. 1). After long-term morphine exposure, the m6A level of TRAF6 mRNA in the vlPAG was significantly increased (Fig. 7). Knockdown of YTHDF1 in the vlPAG inhibited the protein expression of TRAF6 but did not affect the TRAF6 mRNA level (Fig. 7). Our results further suggest that YTHDF1 and TRAF6 are involved in morphine analgesic tolerance and MIH by regulating the inflammatory response after chronic morphine treatments. In addition to TRAF6, YTHDF1 can influence many other mRNAs. Mice with genetic deletion of YTHDF1 (YTHDF1-KO) exhibit learning and memory defects as well as impaired hippocampal synaptic transmission and long-term potentiation. YTHDF1-CLIP analysis showed that YTHDF1 can bind to 1042 mRNAs in the hippocampus [82].

In summary, we found that after chronic morphine treatments, YTHDF1 in the vlPAG was significantly increased and then enhanced TRAF6 protein expression. The upregulation of TRAF6 promotes the expression of inflammation-related factors, such as IL-1β, IL-6, TNF-α and NF-κB, and contributes to the pathogenesis of morphine analgesic tolerance and MIH. Overall, our research results provide novel insight into the molecular mechanisms underlying the inflammatory response in chronic morphine treatments and suggest new therapeutic strategies that might relieve morphine analgesic tolerance and MIH.

## Data Availability

The datasets used during the current study are available from the corresponding author on reasonable request.

## Abbreviations

AAV: Adeno-associated virus
IL-1β: Interleukin-1β
IL-6: Interleukin-6
MIH: Morphine-induced hyperalgesia
MPE: Maximum possible effect
MT: Morphine tolerance
NF-κB: Nuclear factor-κB
OIH: Opioid-induced hyperalgesia
TNF-α: Tumor necrosis factor-α
vlPAG: Ventrolateral periaqueductal gray
WT: Wild-type
